# Advancing Knowledge Translation Through Cultural Adaptation of an Intervention for Latino Family Caregivers of Persons Living With Dementia Using Participatory Action Research

**DOI:** 10.1111/ijn.70143

**Published:** 2026-04-08

**Authors:** Carole L. White, Bianca Shieu, Kimberly S. Peacock, Roxana E. Delgado, Daria B. Neidre, Luis P. Luy

**Affiliations:** ^1^ School of Nursing University of Texas Health Science Center at San Antonio San Antonio Texas USA

**Keywords:** cultural adaptation, dementia, family caregivers, knowledge translation, Latinos, nursing, participatory action research, qualitative research

## Abstract

**Aim:**

The aim of this study is to culturally adapt an evidence‐based intervention for Latino family caregivers of persons living with dementia.

**Background:**

Despite the availability of evidence‐based interventions for family caregivers, Latino caregivers have limited access to culturally relevant interventions.

**Design:**

Qualitative descriptive using a participatory action research approach.

**Methods:**

In 2022, an Advisory Council, comprising Latino family caregivers, was convened to partner with researchers to review and make expert recommendations about cultural adaptations to a caregiver intervention. They also guided the semi‐structured interviews conducted with Latino family caregivers to learn more about culture and caregiving. Data from the interviews were analysed using content analysis. Triangulation of data from the Advisory Council and the semi‐structured interviews was used to identify adaptations to the intervention that would increase its cultural relevance.

**Results:**

Three changes to the intervention content were determined from the different sources of data. The first change was to enhance content about dementia as a medical condition and not just a function of aging. Secondly, given the strong belief among Latinos of their duty to provide care to their elders, a second change was to strengthen the content on self‐care. Finally, given the lower prevalence among Latinos of utilizing palliative care resources, we enriched the information about transitional care resources and their access.

**Conclusion:**

Initially and successfully targeting a specific minority, this study advances the field of knowledge translation by increasing accessibility of an evidence‐based intervention with content that is likely to be relevant for caregivers more widely.

## Summary

What is already known about this topic?

- Provision of appropriate resources is essential to support caregivers in their support role to people living with dementia but Latino family caregivers, particularly, lack such culturally accessible resources.
- There is limited information to guide the process of cultural adaptation of interventions for Latino family caregivers to people living with dementia.

What this paper adds:

- This study demonstrates the practical application of a cultural adaptation framework to adapt a skills intervention for Latino family caregivers.
- This study shows the importance of partnering with caregiver stakeholders to enrich understanding of the cultural context and make relevant adaptations.

The implications of this paper:

- While this study advances the field of knowledge translation through providing detailed information about the process of culturally adapting an intervention specifically for Latino family caregivers, study findings are likely to be relevant and appropriate for wider populations of caregivers.
- Interventions to support family caregivers must be respectful of their diversity and the cultural factors that may influence their caregiving.
- Cultural adaptation of evidence‐based interventions is key to increasing accessibility for the growing number of minority caregivers.

## 1 Introduction

Hispanic/Latino families are disproportionately impacted by dementia, with Latinos 50% more likely than non‐Latino whites to develop dementia (Wu et al. 2016). While Latino caregivers are more likely to provide personal care than non‐Latino caregivers (*Alzheimer's and Dementia* 2023), they tend to use fewer formal services to support their caregiving role (Chow et al. 2010). Latino family caregivers are more likely to report that they lack resources for help or information compared with non‐Latino family caregivers (AARP and National Alliance for Family Caregiving 2020). Furthermore, research suggests that Latino caregivers report poorer health compared with non‐Latino caregivers (Luchsinger et al. 2015; Rote et al. 2019). Despite a robust evidence base of interventions for family caregivers (Cheng and Zhang 2020), few interventions have been translated from research to practice and remain largely unavailable to the millions of caregivers in need of support (Gitlin et al. 2015). This situation is even more grave for Latino caregivers who have limited access to Spanish‐language materials or culturally relevant interventions that can provide information and support them in their caregiving role (*Alzheimer's and Dementia* 2023; Overbaugh et al. 2018; Mehdipanah et al. 2021).

Cultural adaptation of evidence‐based interventions is an important step in knowledge translation, identified as an effective strategy to increase accessibility of interventions for minority populations (Barrera et al. 2017; Torres‐Ruiz et al. 2018). It has been defined as a systematic process of modifying an evidence‐based intervention, which includes but goes beyond language translation to include modifications to curriculum materials and intervention delivery so that it is congruent with cultural meanings and values of the target population (Bernal et al. 2009; Torres‐Ruiz et al. 2018). While systematic reviews have summarized findings of interventions that have been adapted for Latino family caregivers (Dessy et al. 2022; Llanque and Enriquez 2012), the adaptation process has typically not been described in sufficient detail to provide guidance to inform future research in this area. To address this gap and advance the field of knowledge translation, we describe the process of culturally adapting the Learning Skills Together intervention for Latino caregivers to persons living with dementia.

### 1.1 Learning Skills Together Intervention

The care that families provide is becoming increasingly complex, with family caregivers performing tasks that were previously completed by healthcare professionals (Reinhard et al. 2019). Family caregivers report that they find this care difficult to perform and they worry about making a mistake (Burgdorf et al. 2019; Lee et al. 2019; Reinhard et al. 2019). In the context of dementia, care provision becomes even more complex as care recipients may have difficulty understanding instructions and communicating their needs and may resist care (Fauth et al. 2016; Lee et al. 2019). Family caregivers report limited preparation to perform these complex care tasks and, in many cases, report learning by ‘trial and error’ (Reinhard et al. 2019).

In response to this need for training, the Learning Skills Together (LST) intervention was created by a multidisciplinary team of healthcare professionals. This skills‐based intervention focused on the tasks caregivers reported performing most frequently or tasks they reported as difficult, including transfers, medication administration, monitoring co‐morbidities, managing incontinence and nutrition support (Reinhard et al. 2019). Bandura's model of self‐efficacy (Bandura 1997) guided the development of the intervention. Examination of the intervention demonstrated high caregiver satisfaction and improved self‐efficacy (Meyer et al. 2022; Prado et al. 2022).

Based on the pressing need for culturally relevant training resources for Latino caregivers, the next step was to culturally adapt the LST intervention to increase its relevancy and accessibility to the growing number of Latino caregivers. The aim of this paper is to describe the cultural adaptation of the LST intervention.

## 2 Methods

### 2.1 Research Design

This study was undertaken using participatory action research, partnering with Latino family caregivers to examine and culturally tailor the LST intervention. Toward this purpose, we utilized a cultural adaptation framework proposed by Barrera and Castro that uses the sequential steps of information gathering, adaptation design, preliminary adaptation testing and adaptation refinement (Barrera and Castro 2006; Barrera et al. 2013). This paper describes the initial two stages in this framework. Preliminary adaptation testing of the tailored intervention is described elsewhere (White et al. 2025) and adaptation refinement is ongoing. Within this framework, the importance of qualitative research with the target population is emphasized. Accordingly, we undertook a qualitative descriptive study with Latino family caregivers as part of the information gathering stage to increase our knowledge about caregiving among Latino families and to provide a more comprehensive understanding of their culture and caregiving. A Latino Caregiver Advisory Council (LCAC) brought their experience and expertise, providing oversight to the study and partnering with researchers in both the information gathering and adaptation design stages. The goal of the adaptation was to align the LST intervention with values, beliefs and principles congruent with Latino family caregivers while maintaining fidelity to the core elements of the intervention.

#### 2.1.1 Ethics Statement

This study was approved by the University of Texas Health Science Center at San Antonio Institutional Review Board (HSC2022020E). All caregivers participating in the LCAC and the qualitative study provided informed consent prior to participation.

### 2.2 Latino Caregiver Advisory Council

The LCAC comprised five Latino family caregivers (Table 1) and the research team. Caregivers were recruited through Caring for the Caregiver, a program of support for family caregivers. Three LCAC caregivers had previously participated in the LST intervention. A curriculum specialist guided the review of the LST intervention. Three members of the research team had expertise in qualitative research methodology, and all had experience in the field of family caregiving. Monthly meetings were held virtually via Zoom software. Over 9 months, the LCAC was given the responsibility to review the content of each of the six modules, discuss the intervention materials and delivery, and make recommendations for adapting the intervention in a way that they felt would resonate with Latino family caregivers. LCAC caregivers were provided with a stipend for their participation.

**TABLE 1:** Caregiver demographics.

	Age	Gender	Hispanic/Latino/Spanish origin	Education level	Kin relationship to care recipient	ADLs	IADLs
Caregiver participants in Latino Caregiver Advisory Council							
Caregiver 1	72	Male	Mexican American	Bachelor's degree or higher	Wife	Performs all IADLs and assists with some ADLs	
Caregiver 2	74	Female	Mexican American	Some college	Mother	Performs all IADLs and assists with some ADLs	
Caregiver 3	72	Female	Mexican American	Bachelor's degree or higher	Husband	Husband deceased	
Caregiver 4	71	Female	Puerto Rican	Bachelor's degree or higher	Aunt	Living in nursing home (visits daily and assists with care)	
Caregiver 5	66	Female	Mexican American	Bachelor's degree or higher	Husband	Performs all IADLs and assists with some ADLs	
Caregiver participants in semi‐structured interviews							
Caregiver 1	56	Male	Mexican American	Bachelor's degree or higher	Mother	3	7
Caregiver 2	42	Female	Mexican American	Bachelor's degree or higher	Mother	4	7
Caregiver 3	65	Female	Mexican American	Primary level	Husband	1	7
Caregiver 4	58	Female	Mexican American	Bachelor's degree or higher	Mother	1	5
Caregiver 5	81	Male	Mexican American	Secondary level	Wife	5	7
Caregiver 6	79	Female	Mexican American	Secondary level	Husband	6	7
Caregiver 7	66	Female	Mexican American	Some college	Father	6	7
Caregiver 8	42	Female	Mexican American	Bachelor's degree or higher	Mother	0	7
Caregiver 9	63	Female	Mexican American	Bachelor's degree or higher	Mother	6	7
Caregiver 10	63	Female	Mexican American	Secondary level	Mother	5	6
Caregiver 11	64	Female	Puerto Rican	Some college	Mother	5	7
Caregiver 12	80	Male	Mexican American	Some college	Wife	5	7
Caregiver 13	54	Female	Puerto Rican	Some college	Husband	2	7

*Note:* ADLs (Activities of Daily Living – out of 6); IADLs (Instrumental Activities of Daily Living—out of 7).

### 2.3 Qualitative Descriptive Study

#### 2.3.1 Participants

We used purposive criterion sampling to recruit caregivers diverse in kinship relationship to their recipient of care, age and gender. Caregivers were recruited through outreach to our community partners, from the Caring for the Caregiver Program at UT Health San Antonio and referrals from LCAC caregivers. Caregivers were included if they self‐identified as Hispanic/Latino, provided care for a family member or friend with a diagnosis of any form of dementia, and were over 18 years of age.

#### 2.3.2 Data Collection

Consistent with recommendations from Barrera and Castro (2006), we conducted semi‐structured individual interviews with Latino family caregivers to better understand their preferences around culture and caregiving, which could inform the content and delivery of the intervention. A semi‐structured interview guide was developed by the LCAC and included questions about their culture and how they felt it influenced their caregiving role, what tasks they take on in caring for their family member, what they find most challenging in caregiving, and the effect of caregiving on their own health. The interview guide was translated into Spanish by an LCAC caregiver and a research team member.

Interviews were conducted by two members of the research team in English or Spanish, depending on caregiver preference, via Zoom software, and were audio‐recorded. Interviews were conducted over 4 months in 2022 and lasted on average 1 h. The mean age of the 13 caregivers who completed the interviews was 63 years (SD: 12.8); the majority were female (77%), and over half cared for a parent (62%). Most caregivers reported Mexican American heritage (85%), while two caregivers reported Puerto Rico as their country of origin (see Table 1 for caregiver profiles).

#### 2.3.3 Data Analysis

Interviews were transcribed verbatim using the built‐in live transcription tool available through the Zoom software. Content analysis was used to analyse the transcribed data, with comparison across interviews (Gray et al. 2017). Three members of the research team independently reviewed and assigned codes, using an inductive coding approach, to the first transcript. The team then met to discuss their review and the assigned codes and from this review, developed a code book with code definitions, using illustrative examples from the data. Following this team review of the initial transcript, each subsequent transcript was independently reviewed by one team member who then presented the coding to the team for discussion and clarification. The codes along with illustrative quotes from the data were also shared with the LCAC. In coding the last two interviews, no new patterns or significant themes emerged; thus, saturation was reached (Fusch and Ness 2015).

Concurrent with the conduct and analysis of the interviews, the LCAC was meeting regularly to examine each of the six modules of the LST intervention. The LCAC caregivers brought their caregiving expertise to the review of the intervention, identifying areas of content as well as gaps in content where tailoring would enhance the cultural relevancy of the intervention. Subsequently, the full results of the qualitative interviews were shared with the LCAC. The LCAC examined the qualitative data results in light of their observations from reviewing the intervention, with the qualitative data providing confirmation of their observations. The triangulation of the data from these two sources was used to make the recommendations for cultural tailoring of the intervention.

#### 2.3.4 Study Rigour

Credibility of the study was enhanced through the inclusion of a Latino researcher who brought her expert cultural knowledge to the data analysis as well as the extensive knowledge of family caregiving among the members of the research team. The presentation of the codes and illustrative quotations from the qualitative interviews to the LCAC, for their input further enhanced the credibility of the coding process. Methodological triangulation of the two sources of data to inform the recommendations for adapting the intervention provided a more comprehensive picture of caregiving among Latino family caregivers and increased confidence in our results (Heale and Forbes 2013). The development of a code book with clear definitions as well as illustrative quotations, independent coding of transcripts and discussion with the research team, and an audit trail of the decisions made by the LCAC in examining the modules and their subsequent recommendations contributed to the dependability of the results.

## 3 Results

The LCAC confirmed that the intervention addresses topics and skills that are important to the caregiving role and for which there is limited available training, particularly for Latino family caregivers. They underscored the lack of culturally relevant training that is available in Spanish. This was consistent with findings from the interviews where caregivers stated, “we have absolutely no training whatsoever” (Caregiver 4), “It was just, I guess it would be called trial and error” (Caregiver 1). The LCAC made four main recommendations for changes to the intervention, described below and summarized in Table 2.

**TABLE 2:** Cultural adaptations to Learning Skills Together intervention.

Type of change	Reason for change	Changes to intervention
Recommendation 1. Content—Expand content about dementia	Misbeliefs about dementia and its causes, believing that dementia is a normal part of aging.	Add additional information about dementia to the intervention: Enhanced discussion about signs and symptoms of dementia in Week 1; Specific questions added to Facilitator Guide to lead discussion around beliefs about the causes of dementia.
Recommendation 2. Content—Emphasize self‐care through‐out the intervention	Based on the Latino family structure and the importance of family and respect for elders, caregivers may neglect their own self‐care. Need to understand values that are important to Latino family caregivers.	Increased focus on caregiver self‐care and why this is important was added through‐out the intervention: Vignette recorded in English and Spanish with LCAC caregiver discussing the importance of her own self‐care while she cares for her mother; Reflection questions addressing self‐care added to several weekly modules. LCAC recommended that caregivers consider values that underpin the care they provide: LCAC caregivers recorded sound bites in English and Spanish describing values that are important to them in caregiving. These sound bites are introduced Week 1 to open a discussion with participants about values that are important to them in caregiving.
Recommendation 3. Content—Address information gaps and accessibility issues around resources to support advancing care needs	Latino caregivers are more likely to rely on family care rather than formal care including transitional care support such as palliative care, institutional care, and hospice care. Latino caregivers may be fearful and uncertain about hospice care in particular.	Address the need for formal care support, discussing misbeliefs and challenges that may deter caregivers from accessing formal support, in particular palliative and hospice care: In the vignette, LCAC caregiver specifically addresses the importance of a care team and accepting outside support; Additional time added to module on future care planning to specifically address the benefits of palliative and hospice care; Specific questions added to Facilitator Guide to lead discussion about hospice care; Tools for communication with healthcare professionals added to intervention guide; Appendix of formal care resources added to the intervention guide.
Recommendation 4. Presentation and Delivery	Need to reflect the Latino culture in the presentation. Multiple family members may be participating in family caregiving. Importance of relationships.	Template for intervention materials created by the LCAC; Name change to intervention in English and Spanish. Information added to Facilitator Guide (Recruitment Section) about expanding recruitment to target multiple family members to participate in intervention. Additional time added to the first week to learn about participants and begin to build relationships; Additional information added to Facilitator Guide about strategies to connect with participants.

Abbreviation: LCAC (Latino Caregiver Advisory Council).

### 3.1 Recommendation 1: Expand Content About Dementia

The LCAC described a lack of understanding about dementia and its causes among Latino family caregivers, with many families believing that symptoms of dementia are part of normal aging. Caregivers participating in the semi‐structured interviews similarly described misbeliefs about dementia and how these misbeliefs may influence their use of formal support services.

Because still Latino people, still to this day, don't know that it's a disease and you need to get some help so you know how to be able to take care of him. Because it is, it is hard, it's very hard. And, you know, some people just say, Oh, it's just their old age, but it's not just their old age, it has more to it. (Caregiver 7)

The LCAC recommended enhancing the content about dementia in the introduction, including a discussion with caregivers about their beliefs around dementia. They felt that expanding this content in the introduction would improve understanding of later content, particularly related to dementia behaviours.

### 3.2 Recommendation 2: Emphasize Self‐Care Throughout the Intervention

Although the importance of self‐care is implied throughout the intervention, the LCAC felt that the focus should be more intentional, as Latino caregivers often see caregiving as their duty and in turn neglect caring for themselves. They discussed *familismo* (strong family loyalty) and *respeto* (respect for one's elders) as core cultural values that influence caregiving among Latino caregivers. From their experiences, they reported that Latino caregivers are more likely to use family‐based care than formal care services. The LCAC felt that other family members may also share this same expectation around family‐based care, which increases pressure on the family caregiver and leads to feelings of guilt if they engage formal care services. Similar expectations around care provision were described by caregivers participating in the interviews. They described caring for their family as integral to their culture.

You know how Hispanics are like family first, right, and all that stuff. (Caregiver 2)

Taking care of our elderly is part of our culture. It's part of the Latino community. (Caregiver 10)

Caregivers discussed their reluctance to accept outside help.

We don't go out looking for help, either. You know, we want to take care of them. You know, we want to take care of the people that we love and nobody can take care of them like we can. (Caregiver 5)

The LCAC recommended a discussion specifically around formal support services and avoiding caregiver burn‐out. They suggested that it be added to the discussion about self‐care and reinforced throughout the intervention, including weekly reflection questions about how to care for yourself while providing care to a family member. A caregiver from the LCAC recorded a vignette in English and Spanish, discussing the importance of self‐care and creating a care team that helps her to care for her mother.

### 3.3 Recommendation 3. Address Information Gaps and Accessibility Issues Around Resources to Support Advancing Care Needs

The LCAC described a reluctance among Latino caregivers in seeking support for their family members' advancing care needs. They felt that this was related to a lack of information about advanced care resources and how to access them as well as misbeliefs about some of the services. They described a fear of accessing hospice care among many family caregivers, especially in the Latino community. The LCAC also identified a reluctance toward nursing home placement by many in their community. This same reluctance was noted among caregivers participating in the interviews.

So they [Latinos] don't believe too much into taking parents or, you know, grandparents to homes or to care centers. They believe in taking care of their own … my three siblings and I have talked about it and all have agree that you know, we will never take her to a home care center. (Caregiver 11)

Recommendations from the LCAC included the need for specific information about palliative and hospice care as well as a discussion about beliefs and misbeliefs caregivers may have concerning these forms of support as well as institutional care. The LCAC recommended that the content about community resources and how to access resources be expanded. The LCAC stated that members of their community may feel hesitant to express their concerns to healthcare professionals and recommended that specific tools for communication with healthcare professionals be included. Content around a care team was added along with testimonials from caregivers about how they advocate in support of their family members' care needs.

### 3.4 Recommendation 4. Intervention Presentation and Delivery

The LCAC recommended the need for specific changes to the presentation of the intervention and its delivery. They selected the name Aprendiendo Juntos/Learning Together for the adapted intervention and made recommendations for a more colourful template for presentations. To enhance caregiver learning, the LCAC added reflection questions and a practice exercise to each session, highlighting the key points of the content from the week. One LCAC caregiver described this as ‘reflection within their personal space’ and felt that this would contribute to better integration of the content. The LCAC also discussed the family structure and that there may be several family members providing care and recommended recruiting multiple family members to participate in the intervention. This observation was noted in the caregiver interviews.

Four other adults in the household that help me as I need help, all my kids are grown. (Caregiver 1).

We take care, me and my two sisters of my mother. (Caregiver 6)

The LCAC suggested adding additional time to the first session to learn about caregiver experiences, building trust and rapport. Feeling that they are in a supportive learning environment will help build caregiver confidence in providing care for their family member. The LCAC also stressed the importance of being aware of the social context of caregivers who may be participating in the program, including poverty, immigration concerns and racism and how this will compound the complexity of their caregiving situation and their active participation in the intervention.

These recommendations guided the adaptation of the intervention, with changes to both the intervention guide for participants and the facilitator guide. Following this, all materials were translated into Spanish by a professional translator, with independent review by two bilingual members of the research team, one of whom was a family caregiver.

## 4 Discussion

This study describes the practical application of the framework proposed by Barrera et al. (Barrera and Castro 2006; Barrera et al. 2013) in culturally adapting an intervention for family caregivers of persons living with dementia. Researchers often provide little information about the adaptation process including the stakeholders who were involved, how decisions were made, and the resultant changes (Joo and Liu 2021). To advance the field of knowledge translation, there is a need for more detailed information about the process of culturally adapting interventions (Parker et al. 2023; Sidani et al. 2017). Translation of knowledge takes place within a cultural context and thus tailoring interventions to the target population, while maintaining fidelity to the core components of the intervention, can improve intervention acceptability, accelerating the implementation of evidence‐based interventions. Furthermore, there is evidence that culturally adapted interventions may lead to better outcomes for the targeted group (Smith et al. 2011).

We used several strategies to gather information to inform cultural tailoring of the LST intervention. Using a participatory approach helped to ensure that cultural factors related to beliefs and values that are important to Latino family caregivers as well as language translation were addressed in the adaptation. Creating partnerships with the people for whom the research is ultimately meant to benefit enhances the credibility of the research (Jull et al. 2017). The LCAC, composed of family caregivers, guided the adaptation process, reviewing the intervention and the data from the qualitative study to make recommendations for changes that would increase cultural relevance.

The growing number of Latino families affected by dementia underscores the need for accessible evidence‐based interventions that address their diverse needs and circumstances (Massett et al. 2021). While Latino caregivers are very receptive to the idea of training and educational supportive services, reasons for inaccessibility include language barriers and limited cultural acceptability (Sehar et al. 2023). Interventions or programs that are culturally insensitive or irrelevant may discourage caregivers from seeking support (Overbaugh et al. 2018). The LCAC confirmed the importance of culturally tailored programs. They identified several local initiatives aimed at addressing the needs of Latino family caregivers, such as Spanish language education programs provided by the Alzheimer's Association, the Hispanic/Latino Caregiver Council that brings together family caregivers as well as members of Hispanic‐serving community‐based services, and the Caring for the Caregiver program, an integrated service and research program based in a School of Nursing.

While these local initiatives address specific community needs, our findings suggest that the experiences of Latino caregivers have broader applicability beyond this specific cultural group. Aligned with a recent systematic review on East Asian dementia caregivers (Wang et al. 2023), our results highlight how cultural mandates serve as a double‐edged sword. Consistent with our separate investigation (Shieu et al., under review), familism fostered pride and unity, yet simultaneously reinforced restrictive gendered roles and created barriers to formal support. Specifically, seeking outside help was often perceived as a failure of duty. This dynamic closely mirrors the concept of *filial piety* in East Asian societies, which validates caregivers through social honour but imposes immense pressure and isolation, particularly on women (Wang et al. 2023). It is also important to note the relevance of these findings beyond minority populations to female daughters in general who still account for the majority of caregivers, often juggling care for parents, care for their own children and employment (AARP and National Alliance for Family Caregiving 2020). These parallels highlight that while cultural nuances exist, the fundamental need for dementia education and supportive interventions is universal for families navigating the complexities of caregiving.

### 4.1 Strengths and Limitations

A strength of this study is the multiple approaches used to gather information and triangulating these data to inform the cultural adaptation of the LST intervention. Data triangulation increases the credibility and validity of the results. Another strength is the participatory research approach, partnering with Latino family caregivers and bringing their experiences and expertise to guide the adaptation process. Three caregivers serving on the LCAC had previously participated in the LST intervention. Their direct experience with the intervention was particularly valuable in identifying areas for cultural adaptation.

Although a purposive sampling approach was used to recruit a diverse sample of caregivers, a limitation is that those caregivers who agreed to participate are more likely to represent caregivers who may be more confident in their caregiving role and more able to access resources. There may, therefore, be aspects of the LST intervention that require further adaptation to ensure accessibility to the larger population of Latino family caregivers. We acknowledge a limitation in the translation of the participant and facilitator intervention guides which did not undergo forward and backward translation. The translation of the materials, however, was reviewed by a Latino family caregiver to support the cultural relevancy of the translation. Finally, the next step required for this research will be to pilot test the adapted intervention and identify any additional modifications needed to enhance the efficacy of the intervention.

## 5 Conclusion

Care and support provided to family caregivers must be respectful of their diversity and of the cultural factors that may influence their caregiving. Through the research presented here, several concepts important to Latino family caregivers such as personalismo and familismo were identified. In selecting evidence‐based interventions to be implemented in the routine care for family caregivers, it is important that the intervention be culturally relevant to the target population. Future research is needed to better understand the value of culturally adapted interventions, including their efficacy and cost‐effectiveness. Few studies have compared a culturally adapted program to one that has not been adapted.

Cultural adaptation of an evidence‐based intervention is an important aspect of increasing knowledge dissemination across diverse populations. A methodology that gathers information to identify contextual factors influencing uptake of the intervention and making adaptations will help to ensure equity in supporting family caregivers.

Our study has broader implications for the nursing profession. The research presented here is a result of the growing collaboration between a School of Nursing and the community to conduct research that is building evidence for best practice to support family caregivers, with a focus on Latino family caregivers. Addressing family caregiver needs is an expected competency of the professional nurse (American Association of Colleges of Nursing 2021). In identifying interventions to support family caregivers, it is important that nurses appraise the quality of the evidence, including best practice in cultural adaptation of interventions. The methodology presented here, using a framework to guide the adaptation, can influence future nursing research and translation of evidence into practice.

## Author Contributions

C.L.W. designed the study, analysed data and prepared the manuscript. B.S. reviewed and edited the manuscript. K.S.P. analysed data and edited the manuscript. R.E.D. reviewed and edited the manuscript. D.B.N. and L.P.L. collected and analysed the data. All authors approved the final version for submission.

## Funding

This work was supported by RRF Foundation for Aging, 2021318.

## Conflicts of Interest

The authors declare no conflicts of interest.

## Data Availability

The data that support the findings of this study are available on request from the corresponding author. The data are not publicly available due to privacy or ethical restrictions.
